# The potential role of exercise in mitigating fertility toxicity associated with immune checkpoint inhibitors (ICIs) in cancer patients

**DOI:** 10.1186/s12576-024-00950-3

**Published:** 2024-11-30

**Authors:** Parivash Jamrasi, Mia Tazi, Nur Afiqah Zulkifli, Jun Hyun Bae, Wook Song

**Affiliations:** 1 Department of Physical Education, Seoul National University, Seoul, Republic of Korea; 2 Faculty of Life Sciences and Medicine, King’s College London, London, UK; 3 Institute of Sport Science, Seoul National University, Seoul, Republic of Korea; 4 Institute On Aging, Seoul National University, Seoul, Republic of Korea

**Keywords:** Immunotherapy, Fertility, Exercise, Immune checkpoint inhibitors, Oncofertility

## Abstract

Over the last decade, therapeutic advances in cancer immunotherapy have rapidly progressed, leading to an expansion of clinical trials and the development of novel immune checkpoint inhibitors (ICIs) and combination treatments. While ICIs offer substantial clinical benefits, they are also associated with various side effects, notably concerning endocrine function and potential gonadal damage following the initiation of immunotherapy. Exercise has demonstrated promise in enhancing treatment efficacy, including symptom reduction in cancer patients. Research has also established the benefits of exercise in managing fertility and reproductive health. However, there is limited data on the effectiveness of exercise in mitigating fertility-related side effects specifically in patients undergoing ICIs therapy. Given that a significant number of cancer patients are of reproductive age, it is crucial to address potential sexual side effects and offer fertility preservation options. Ensuring that patients are well-informed and supported in their reproductive health decisions is vital. This review reports the prevalence of immune-related adverse effects linked to fertility in cancer patients undergoing ICIs, explores the potential mechanisms by which ICIs may impact reproductive health, and emphasizes the role of exercise in mitigating these adverse effects.

**Graphical Abstract:**

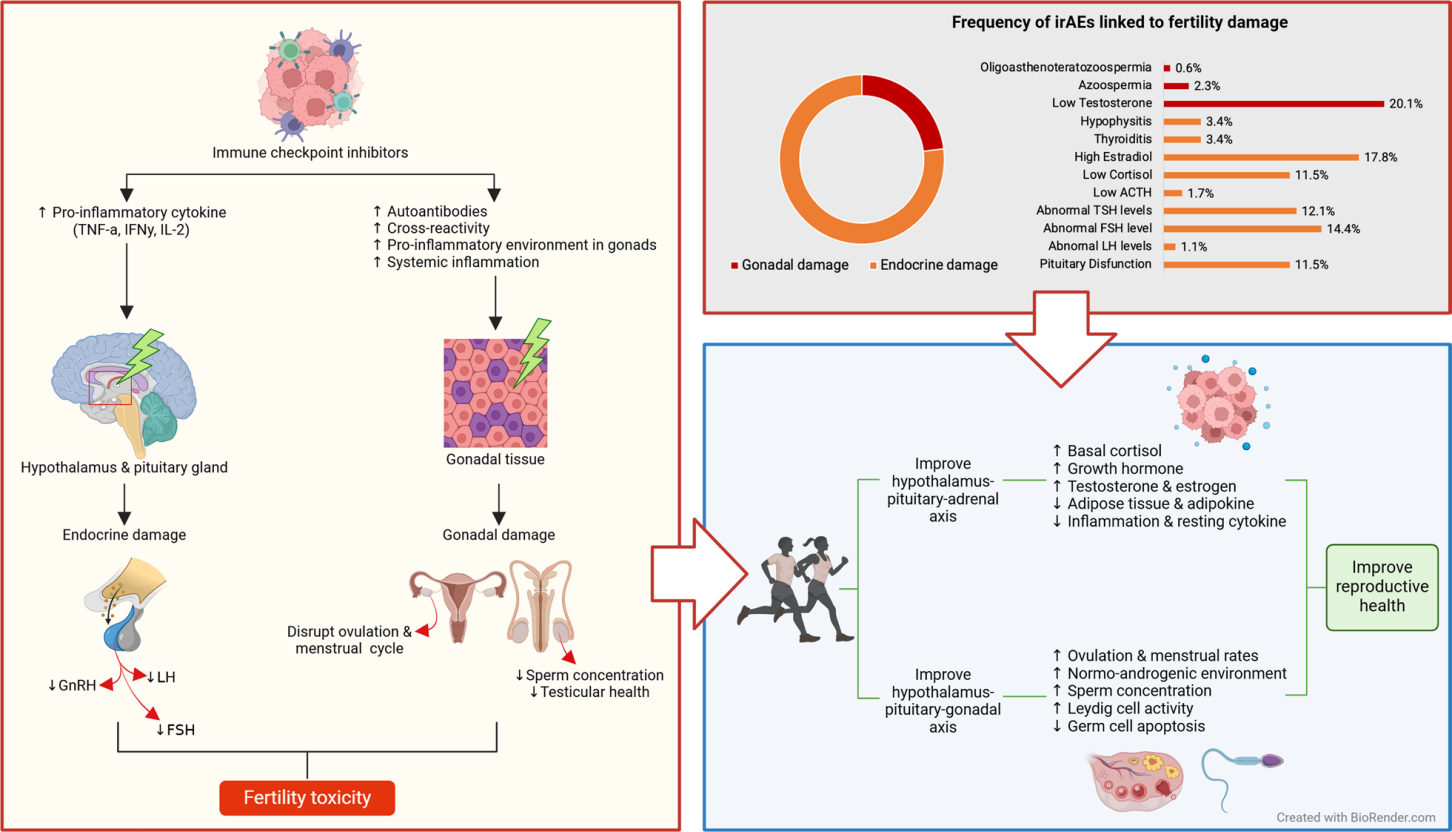

## Background

In recent years, therapeutic advances in cancer immunotherapy have rapidly emerged, reflecting the importance of the interaction between the human immune system and cancer [1]. Immunotherapy is primarily employed to bolster the immune system by modulating the immune microenvironment, enabling immune cells to target and eliminate tumor cells at several critical points [2]. Currently, immunotherapeutic agents, such as immune checkpoint inhibitors (ICIs), are gaining significant attention [3].

ICIs, also known as immune checkpoint blockade therapy, targeting cytotoxic T-lymphocyte associated protein 4 (CTLA-4), programmed cell death protein 1 (PD-1), and programmed death-ligand 1 (PD-L1) prevent these receptors and ligands from binding, thereby disrupting signaling that T cells can recognize and attack cancer cells [4]. The use of ICIs is rising exponentially, with approximately 40% of cancer patients in the United States eligible for therapy with ICIs in 2019. Additionally, clinical trials of immunotherapy continue to expand with the development of novel ICIs agents and combination treatments [4].

While the success of these therapies is well-documented, ICIs are associated with significant immune-related adverse effects (irAEs) due to their distinct mechanism of action, which differs from that of cytotoxic chemotherapy [3, 5, 6]. As a result, there should be a heightened awareness that any new symptoms may be related to the treatment [4]. Recent studies have highlighted endocrine disrupts and hormone-related changes linked to primary and secondary hypogonadism, among other data [7–12]. Given that a significant number of cancer patients are of reproductive age, it is estimated that approximately 30% of malignant melanomas, 15% of colorectal carcinomas, 10% of lung and renal cancers, and 5% of bladder and prostate cancers occur in individuals under 50 years old [13]. Consequently, it is essential to address potential sexual side effects and offer guidance on fertility preservation to ensure that patients are well-informed and supported in making decisions about their reproductive health.

Considering the role of exercise in cancer treatment, regular exercise is well-documented to mitigate cancer progression by influencing metabolic and immune functions, affecting the tumor microenvironment (TME), enhancing drug delivery to cancer lesions, and improving the efficacy of anticancer therapies [14]. While ICIs can adversely affect fertility, emerging evidence suggests that regular exercise may help mitigate these effects. Research has established significant benefits of exercise in managing fertility by influencing both the hypothalamic–pituitary–adrenal (HPA) axis and the hypothalamic–pituitary–gonadal (HPG) axis. This regulation leads to lower gonadotropin levels, improved immunity, and reduced release of sex hormones and inflammation [15–18].

While these findings underscore the importance of incorporating exercise into fertility management strategies, there is a significant gap in clinical research on the impact of exercise on the reproductive health of cancer patients treated with ICIs. This highlights the need for specific studies that focus on exercise as a primary intervention for reproductive health, given its potential to mitigate other adverse effects of cancer treatment.

### Rationale

Despite the recent surge in immunotherapy, little data are reporting on the effect of exercise alongside immunotherapy in mitigating fertility-related side effects. This review aims to highlight the potential mechanisms of ICIs on the reproductive health of cancer patients and outline the benefits of exercise in mitigating this adverse effect. Possible mechanistic reasons for why exercise could be beneficial as an intervention and guidance for future studies will be discussed.

## Mechanisms of ICIs in tumor suppression

**Fig. 1:**
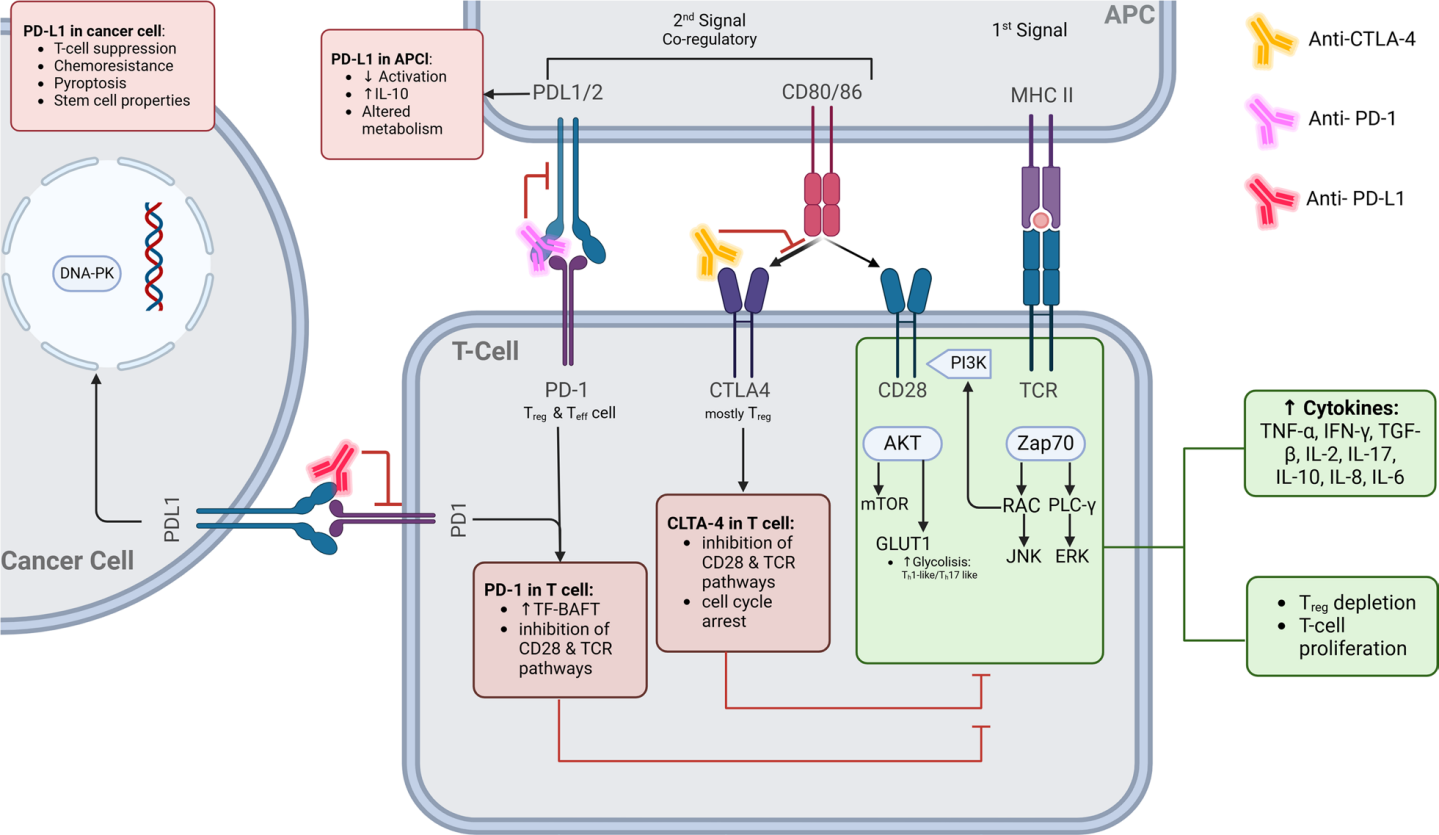
Mechanisms of approved immune checkpoint inhibitor drugs in tumor suppression. Lymphocyte T cells, including helper T cells (Th-cells/CD4 +), require two signals for activation: the first involves T cell receptor (TCR) interaction with antigen-presenting cells (APCs) through the major histocompatibility complex (MHC) Class II, and the second involves co-regulatory signals. CTLA-4, an inhibitory receptor primarily on Tregs, competes with CD28 for binding to APCs’ CD80/CD86, leading to immune suppression. Anti-CTLA-4 therapy blocks this interaction, enhancing T-cell activation through pathways involving ZAP70, AKT, and PI3K, and depleting Tregs, which increases cytokine elevation and promotes an anti-tumor immune response within the tumor microenvironment (TME). Cancer cells exploit PD-1 binding through PD-L1 to evade immune detection, inhibiting T-cell activity and facilitating tumor growth. PD-1 signaling in T cells leads to increased TF-BAFT expression, inhibiting CD28 and TCR pathways, and resulting in T-cell exhaustion. Anti-PD1 antibodies block this interaction, restoring T-cell activation and immune response against cancer. PD-L1, expressed on various cells including cancer cells, can translocate to the nucleus and interact with DNA-dependent protein kinase (DNAPK), promoting cell survival, chemoresistance, and stem cell properties. Anti-PD-L1 therapies prevent PD-1/PD-L1 binding, enhancing T-cell activation and creating a pro-inflammatory environment conducive to anti-tumor activity. Created with https://www.BioRender.com

Understanding the mechanisms underlying the ability of ICIs to modulate cell interactions, which can enhance the body’s immune response against cancer cells, can lead to improved patient outcomes and reduce the effect of irEAs on fertility. Considering the approved ICIs drugs, the mechanisms of action are detailed in four distinct sections: anti-CTLA-4, PD-1, PD-L1 and combination therapies, as depicted in Fig. 1.

### Anti CTLA-4 pathways

CTLA-4, an inhibitory receptor primarily on regulatory T cells (Tregs), suppresses cytokines production and causes cell cycle arrest after T cell receptor (TCR) interaction. In cancer immunotherapy, anti-CTLA-4 antibody inhibits the binding of CD80/CD86 to CTLA-4, therefore binding to CD28 on Treg cells [19]. CD80 and CD86 are surface glycoproteins on APCs that bind to both CTLA-4 and CD28, making them competitive receptors. While CD28 is more widely expressed, its binding to CD80/CD86 is weaker than that of CTLA-4, making CD80/CD86 more likely to interact with CTLA-4 when present [20, 21]. Two mechanisms have been proposed for Anti-CLTA-4 therapy: targeting intratumoral Tregs and targeting TME Tregs [19]. Anti-CTLA-4 favors anti-tumor activity by blocking CD80-CTLA-4 binding which increases T-cell activation leading to clonal expansion [22]. Anti-CTLA-4 also leads to antibody-mediated Treg depletion through its interaction with Fc*γ* receptors (Fc*γ*Rs) on innate cells which lead to perforin and granzyme expression [23]. The depletion of Treg cells allows for less downregulation of the immune system. Within the TME, the metabolism of Treg cells can change from oxy-phosphorylative to glycolytic, giving it the properties of Th-1 like Tregs or Th-17 like Tregs (this depends on the cytokines present) [24, 25]. The Th-like Treg cells then express their pro-inflammatory cytokines and immune response leading to increased levels of interleukins (IL-1, IL-2, IL-6, IL-8), tumor necrosis factor-alpha (TNF-*α*), and interferon-gamma (IFN-*γ*) in patients after ICIs treatment [24]. This increased immune response allows for T cells to kill cancer cells.

### Anti-PD-1 and anti-PD-L1 pathways

PD-1, an inhibitory receptor activated by PD-L1/L2, is found on various immune and cancer cells. In T cells, it reduces activation, proliferation, cytokine production, and alters metabolism, leading to T-cell exhaustion. The role of PD-1 is to balance the immune response by preventing excessive responses and by maintaining immune tolerance [26]. Unlike CTLA-4, PD-1 on T cells requires the major histocompatibility complex (MHC) proteins to be presented by the same cell as PD-1 ligands for its co-stimulatory signal to be activated. These cells include antigen-presenting cell (APCs) and tumor cells. Beyond inhibiting stimulatory pathways, such as Zap70, PD-1 increases the expression of the transcription factor BATF, causing it to counter effector transcriptional programs [27]. In addition to T-cell exhaustion, the PD-1 pathway plays a crucial role in regulating various immune responses, including humoral response, T follicular helper (TFH), follicular regulatory T (TFR) cells levels, and Treg cell levels, due to its expression on multiple cell types [28]. PD-1 is used by cancer cells to regulate the immune response by binding to it through PD-L1 and inhibiting its ability to recognize and attack cancer cells. This allows cancer to continue to grow and spread without being targeted by the immune system. Anti-PD-1 antibody prevents PD-L1 from binding which in turn affects the aforementioned regulation. This allows for upregulation of the immune response, which either limits cancer growth or promotes the destruction of cancer cells [28].

Ligand PD-L1 is expressed on a larger variety of cells than PD-1, such as immune cells, cancer cells, and some non-hematopoietic cells. Its expression is usually upregulated from inflammatory cytokine production [29]. PD-L1 can bind with PD-1 and CD80. The physiological role of PD-L1 is central/peripheral tolerance, immune exhaustion, and regulation of the anticancer immune response [30]. In cancer cells, PD-L1 can translocate to the nucleus and act as a stimulatory signal that activates proliferation and survival pathways such as chemoresistance and transcription regulation [31, 32]. Similarly to anti-PD-1, anti-PD-L1 aims to block the PD-1/PD-L1 binding thereby allowing T-cell activation and a pro-inflammatory environment leading to anti-tumor response [33].

### Combination therapy pathways

The effects of combination therapy are not the same as ICIs taken separately. Combination therapy with anti-CTLA-4 and anti-PD-1 checkpoint blockade brings about changes at the cellular level. These changes can result in enhanced activation and proliferation of effector T cells, a reduction in regulatory T cells, and a more inflamed and immune-active tumor microenvironment. Additionally, combination therapy induces unique gene expression profiles and enhances memory T cell responses, contributing to a more robust and long-lasting anti-tumor immunity [34, 35].

## Potential mechanisms of immune-related adverse effects on fertility

While the impact of traditional cancer treatments on this system is fairly well understood, the effects of immunotherapy might not be as clear. Despite research into the side effects of ICI treatments [36], there is an absence of studies specifically focusing on fertility issues [7]. Though the upregulated immune response caused by ICIs is beneficial in the TME, it can lead to irAEs both in proximal and distal organs of the tumor. These mechanisms related to fertility issues can be categorized under endocrine damage or gonadal damage.

### Endocrine damage

Increased cytokines levels from ICIs can cross the blood–brain barrier (BBB) and affect the hypothalamus and pituitary gland [37]. These cytokines can impact the function and hormone production of the hypothalamus. Blocking CTLA-4 using monoclonal antibodies in humans may increase CD4 + and CD8 + T cell levels and lead to the release of cytokines, such as TNF*α*, interferon-*γ* (IFN*γ*), and IL-2 [21, 38]. TNF-*α* is an example of a cytokine that can signal the reduced secretion of gonadotropin-releasing hormone (GnRH) [39]. This, in turn, can impact reproductive function given its impact on general hormone production and signaling through the hypothalamus–pituitary–organ (HP(X)) axis. Beyond the hypothalamus, ICIs produced cytokines can act on signaling mechanisms to the pituitary gland [40] and further along the axis, with the adrenal gland and thyroid where these cytokines generally produce an inhibitory effect [41, 42].

Hypophysis is an inflammation of the pituitary gland that can lead to hormonal imbalances due to its impaired function [18]. The pituitary gland acts on the ovaries and testes, and affects reproduction function in females and males through two main hormones: luteinizing hormone (LF) and follicle-stimulating hormone (FSH) [43]. The HP(X) axis can be directly affected by anti-CTLA-4. CTLA-4 is highly expressed on the anterior pituitary tissue allowing the antibody to directly bind to it. Anti-CTLA-4 could cause hypophysitis. Due to the pituitary’s role in producing key sex hormones as well as adrenal and thyroid function, this can, directly and indirectly, impact fertility and may lead to hypogonadotropic hypogonadism [5, 40]. This form of hypogonadism is a condition where the gonads produce insufficient sex hormones, leading to delayed puberty, irregular menstrual cycles, and infertility [44].

ICIs can also lead to the production of pathogenic antibodies given the upregulated immune system due to Treg depletion. Anti-CTLA-4 and anti-PD-1 upregulate B cell quantities leading to augmented T cell–B cell interactions which may result in the production of autoantibody production [45, 46]. Given this information, patients with gonad-related tumors could develop auto-antibodies targeting healthy gonad tissues, thus impacting fertility. One example of autoantibody irAE indirectly affecting fertility is diabetes type 1. In this case, the pancreas beta cells are affected and end up showing similar symptoms to non-ICI diabetes type 1 patients; the imbalanced leptin levels signal an energy-deficient state [47]. This can suppress the HPG axis, leading to reduced secretion of gonadotropins (LH and FSH) [47]. This suppression can also result in hypogonadotropic hypogonadism. Chronic high blood sugar from type 1 diabetes can lead to glucose toxicity and the formation of advanced glycation end-products (glycation of proteins and lipids due to sugar exposure) [48], which may damage ovarian follicles, leading to early menopause. Subcutaneous insulin injections for type 1 diabetes may cause high insulin levels, which promote androgen production and may contribute to PCOS [47]. Increased TNF-alpha whose receptor is TNFR1 can lead to a pro-inflammatory response including monocyte infiltration, polarization of pro-inflammatory macrophages, tissue inflammation which may impact insulin sensitivity and glucose levels given their impact on the IRS1/2 complex [49]. This can be a precursor to type 2 diabetes mellitus by causing impaired glucose tolerance. Type 2 diabetes affects fertility to similar degrees of diabetes type 1 [50]. However, the mechanism remains different with type 2 diabetes leading to oxidative stress from systemic inflammation in gonads and type 1 diabetes through altered epididymal voiding [50]. Patients with genetic predispositions for impaired glucose tolerance [51], beta cell failure [52], and in chronic energy imbalance could be at increased risk to becoming impaired glucose tolerance given the increase systemic inflammation.

### Gonadal damage

Cross-reactivity in immunology refers to the phenomenon where immune cells, particularly T cells, recognize and respond to antigens that are structurally similar but derived from different sources, such as pathogens, tumor cells, and normal tissues. [5]. This can have significant implications for gonadal tissues and fertility, particularly in the context of hypogonadism. Primary hypogonadism is caused by dysfunction inside the gonads, whereas central hypogonadism results from abnormal changes in the hypothalamus–pituitary reproductive axis. Low sex hormone levels (testosterone in males and estradiol in women) and variable gonadotropin levels (FSH and LH) are biochemical markers of hypogonadism. Clinically, it can result in erectile dysfunction and non-obstructive azoospermia in males, and anovulation and amenorrhea in women [53].

Cross-reactivity can lead to immune responses against self-antigens in the gonads. For instance, during infections or in autoimmune diseases, antibodies or T cells that target foreign antigens might also recognize similar antigens in testicular tissues. This can trigger inflammation and tissue damage, potentially compromising the blood testis barrier (BTB) [54]. In the normal testis, the BTB limits the interaction between germ cell antigens and interstitial immune cells. Secretion of pro-inflammatory cytokines acts on adherents and tight junctions, altering the BTB permeability. [54] The BTB is crucial for protecting germ cells from immune system attack, and its disruption can expose germ cells to harmful immune reactions.

Testes are considered an immunoprivileged organ because of the mechanisms that protect it from being attacked by the body’s immune system by creating an immunosuppressor microenvironment [55]. However, the testis can experience autoimmune reactions if infection or inflammation overwhelms its immunosuppressive mechanisms which can occur with ICIs due to the secretion of pro-inflammatory cytokines and reduced ability of Tregs [56]. This disrupted microenvironment can lead to germ cell apoptosis and testicular damage, resulting in aspermatogenesis and infertility [54].

Systemic inflammation and other irAEs can affect fertility in indirect ways. Fever caused by other adverse effects such as tuberculosis or pneumonitis can affect reproductive function in both short and long-term ways [57]. This is especially studied in men where sperm quality and quantity can be affected [58]. Systemic inflammation can also lead to a pro-inflammatory environment of gonads which has been linked to reproductive ageing in female mice [59]. with ovaries being considered a key interaction organ between the immune and endocrine system [42], this inflammation can also affect the ovulation cycle given the importance of inflammatory-related events of ovulation include the recruitment. Inflammation homeostasis is important for ovarian cycle [60].

## Prevalence of fertility issue in cancer patients treated with ICIs

Over the last decade, studies have increasingly documented reports of endocrine dysfunction following the initiation of immunotherapy. However, there are limited clinical data on the effects of ICIs on human reproductive functions. A comprehensive analysis of the endocrine toxicity spectrum of ICIs has revealed 6089 cases of associated endocrinopathies, for both male and female patients, regardless of cancer status [61]. Accordingly, significant associations were identified, highlighting both common endocrinopathies (thyroid dysfunction, hypophysitis, adrenal insufficiency, type 1 diabetes mellitus (T1DM)) and rare endocrinopathies (hypoparathyroidism, diabetes insipidus, hypogonadism) [61]. A meta-analysis of 110 studies including randomized trials, prospective, and retrospective studies revealed that PD-1/PD-L1 inhibitors, particularly nivolumab, and pembrolizumab which are both categorized under PD-1, are associated with a high incidence of thyroid dysfunction, especially hypothyroidism[12]. Additionally, combination therapy increases the incidence of various endocrine adverse events, including hypothyroidism, hyperthyroidism, hypophysitis, and primary adrenal insufficiency [12]. However, the onset time of endocrinopathies varied between different ICI therapies, typically occurring within 12 weeks for anti-CTLA-4 monotherapy but ranging from 0 to 48 weeks for anti-PD-1 monotherapy [61].

In order to find insights on possible fertility damage from ICIs, a search protocol was set up on Medline, Web of Science, and Embase to extract evidence on reported irAEs from the past 10 years (2014–2024). After eliminating duplicates and screening 4945 articles, 81 relevant research papers were selected. The inclusion criteria for paper selection were studies published in accurate journals in the form of scientific papers that reported on the pituitary–gonadal axis and endocrine levels with potential effects on reproductive health in cancer patients undergoing ICIs treatment (during treatment or follow-up). For female participants, the fertility age was set to those under 50 years, whereas no age limit was applied for male participants. Studies that included data from postmenopausal women or non-cancer patients in the outcomes were excluded from this review to maintain the integrity and relevance of our findings. However, studies that reported outcomes separately by gender were included. Taking into account the inclusion and exclusion criteria, ultimately 11 studies were identified that reported outcomes related to adverse events associated with ICIs treatment, linked to hypogonadism. As presented in Table 1, both primary and secondary hypogonadism were observed across different cancer types and ICIs regimens.

**Table 1:** Evidence of reported irAEs associated with fertility damage in cancer patients treated with ICIs

Authors	Year, Country	Participants	Median age	ICI treatment	Sample size (*n*)	irAEs linked to fertility
Jessel, Shlomit, et al. [62]	2022, USA	Melanoma and renal cell carcinoma patients	64 years	Ipilimumab + nivolumab, ANTIPD-L, anti-PD-L1 + investigational agent,	*n* = 20, male	Low testosterone (*n* = 15)
Peters, Madeline, et al. [63]	2021, USA	Malignant melanoma patients	64 years	Pembrolizumab, ipilimumab, nivolumab	*n* = 49, male	Low testosterone (*n* = 34), hypophysitis (*n* = 4), pituitary dysfunction (*n* = 19), Adrenal insufficiency (*n* = 16), thyroid dysfunction (*n* = 18)
Salzmann, Martin, et al. [64]	2021, Germany	Cutaneous malignancies or uveal melanoma patients	49 years	Pembrolizumab, nivolumab or/and ipilimumab	*n* = 25, male	Azoospermia (*n* = 3) oligoasthenoteratozoospermia (*n* = 1), hypophysis (*n* = 5), thyroiditis (*n* = 6)
Tulchiner, Gennadi, et al. [65]	2021, Austria	Metastatic renal cell carcinoma patients	61.5 years	Nivolumab	*n* = 12, male	Low FSH, high Estradiol, high LH/FSH
Lindner, Andrea Katharina, et al. [66]	2023, Austria	Metastatic urothelial patients	70 years	Pembrolizumab	*n* = 18, male	High estradiol, low testosterone, high FSH

irAEs, immune-related adverse events; TSH, thyroid stimulating hormone; FSH, follicle-stimulating hormone; Free T4, free thyroxine; ACTH, adrenocorticotropic hormone; LH, luteinizing hormone; PD-L1, programmed death-ligand 1

The presented studies in Table 1 included patients diagnosed with various types of cancer, such as Ewing sarcoma, renal cell carcinoma, lung adenocarcinoma, malignant melanoma, cutaneous malignancies, urothelial carcinoma, and advanced gastric cancer treated with various ICIs, including CTLA-4 Inhibitor (ipilimumab drug), PD-1 inhibitors (toripalimab, nivolumab, and pembrolizumab drugs), and PD-L1 inhibitors (atezolizumab drug) highlighting a range of cancer types and treatments associated with hypogonadism and other endocrine disorders. While most reported cases are in males (98.45%) there is limited evidence regarding females (1.55%) before menopause. Accordingly, low testosterone was the most frequently reported irAE in cancer patients who received ICIs treatments, followed by thyroid dysfunction. One study reported a case where a patient had a normal spermiogram before ICIs treatment but developed azoospermia one year after initiation of treatment [64]. Additionally, testosterone suppression was reported in patients who developed low testosterone after having normal testosterone levels prior to starting ICIs immunotherapy [63]. Though none of the studies specifically commented on the infertility or sexual side effects of these drugs, a trend was noted between testosterone levels and both adrenal dysfunction and thyroid dysfunction [63]. Although studies on irAEs affecting fertility in patients treated with ICIs are limited, case reports provide valuable insights. Wang et al. (2024) reported subclinical hypothyroidism in a male Ewing sarcoma patient treated with anti-PD-L1 [67], while Chang et al. (2019) documented low FSH and hypothyroidism in a female lung adenocarcinoma patient on nivolumab [68]. Lupi et al. (2019) and Rabinowitz et al. (2021) described hormonal imbalances and azoospermia, respectively, in melanoma patients treated with nivolumab and ipilimumab [69, 70]. Additionally, Gunawan et al. (2018) reported a male patient with hypophysitis and anterior pituitary dysfunction, which severely disrupted testosterone and other hormone levels, leading to potential fertility impairment [35]. Taken together, these studies suggest that ICIs may cause disruptions in the pituitary–gonadal axis and endocrine function, which can adversely affect reproductive health.

## Role of exercise in ICIs treatment for cancer patients

The connection between exercise and cancer suppression is complex, engaging both metabolic and immune pathways, with myokines being key players [71]. Myokines have direct anticancer effects, including inhibiting cell proliferation and promoting apoptosis, as well as indirect effects through the enhancement of metabolic and immune responses; and regular exercise supports these processes, establishing an environment that is less conducive to tumor growth while enhancing the body’s capacity to eliminate cancer cells [71].

Several myokines, including IL-6, irisin, oncostatin M (OSM), secreted protein acidic and rich in cysteine (SPARC), and decorin, which increase during exercise, play critical roles in cancer suppression by reducing cell proliferation, promoting apoptosis, and inhibiting metastasis [14, 71, 72]. Additionally, myokines like IL-6 and IL-15 enhance immune cell function, promoting NK and T cell proliferation [14, 73]. IL-6 also aids in lipolysis, increases insulin sensitivity, and reduces adipose tissue macrophage accumulation, all of which contribute to mitigating cancer progression [74]. Overall, myokines also help improve,, insulin resistance and lower chronic inflammation, further aiding cancer suppression. In addition to their metabolic and immune-regulating roles, myokines also influence the TME by enhancing vascular function. The TME, particularly the immune microenvironment, is crucial for cancer control. Exercise-induced myokines like IL-6 facilitate the mobilization of NK cells into tumor tissues, thereby improving immune surveillance and inhibiting tumor progression [74]. This bolstered immune response can also enhance the effectiveness of immunotherapies, as seen in preclinical studies where exercise improved the efficacy of immune checkpoint inhibitors. For instance, in preclinical cancer models, exercise increased CD8 + T cell activity and infiltration into tumors, amplifying the effects of PD-1 inhibitors and immune checkpoint blockade [75, 76].

Resistance and aerobic exercise lead to a multitude of physiological adaptations with their impacts raging to muscular strength/size, substrate metabolism and hemodynamics [77]. The practice of exercise not only alleviates pro-inflammatory effects, but also exhibits anti-inflammatory effects thus reducing systemic inflammation [78]. The role of exercise during cancer care is initially based on these principles and is prescribed at various stages of treatment. The effect of physical activity has shown promise on the following outcomes: efficacy of such as reduction of some symptoms of cancer [79], palliative care on quality of life [80], and reduction of the side effects of cancer treatments [14]. Hojman (2018) further explains the role of exercise and the potential molecular mechanisms behind it [14]. On top of the immune system modulation, the review highlights tumor intrinsic effects (intratumoral signaling) being the main mechanisms to influence exercise’s effect on cancer. Intratumoral signaling refers to the communication processes that occur within a tumor. Through exercise, this signaling can be modified through physical and endocrine effects, which impact blood flow, vascular shear stress, pH regulation, heat production, and sympathetic activation. The sum of these changes effects many aspects of the tumor often leading to reduced growth and expression [14]. Despite these positive effects, there is a notable absence of clinical research on the impact of exercise on the reproductive health of cancer patients treated with ICIs, highlighting the need for targeted studies that explore exercise as a primary intervention to address treatment-related adverse effects.

## The potential impact of exercise on reproductive health

The benefits of exercise in managing fertility have been successfully established by research on physical exercise and reproductive health. A previous review study by Xie, Fangfang, et al. (2022) mentioned that exercise can prevent infertility by influencing the hypothalamic–pituitary–gonadal axis, which lowers gonadotropin levels, boosts immunity and prevents the release of sex hormones and inflammation [15]. Another study highlighted that body mass index (BMI) can negatively impact fertility in both males and females, and exercise has been proven to improve this condition through weight-loss lifestyle intervention [81].

Various exercise modalities and intensities have proven to have a positive impact on male reproductive markers [82]. After 24 weeks of exercise training, moderate-intensity continuous exercise was proven to be more beneficial in improving sperm concentration, counts, motility, and morphology (*p* ¡ 0.05) compared to high-intensity continuous exercise and high-intensity interval training [82]. On the other hand, anti-mullerian hormone, a clinical marker of ovarian reserve, was proven to be lower in women with a lack of physical activity (*p* = 0.025) and vigorous physical activity (*p* = 0.039) [83]. This suggested that moderate-intensity physical exercise may improve infertility issues in both males and females.

To add, different exercise intensities have distinct effects on the immune system. Moderate-intensity exercise is frequently cited as the most effective for enhancing the immune system, whereas high-intensity exercise may lead to downregulation of immune responses. On the other hand, low-intensity exercise is often recommended for beginners and individuals with clinical conditions, as it offers a safer, accessible approach to physical activity [84]. On the other hand, in the 1990s, Dr Nieman proposed the “J-shaped hypothesis”, suggesting that exercise intensity impacts immune function in a J-shaped curve. According to this idea, moderate exercise can improve immune health compared to being inactive, while high-intensity exercise may depress the immune system [85]. This understanding aligns with the open window theory, which suggests that intense exercise can temporarily reduce immunity.

**Fig. 2:**
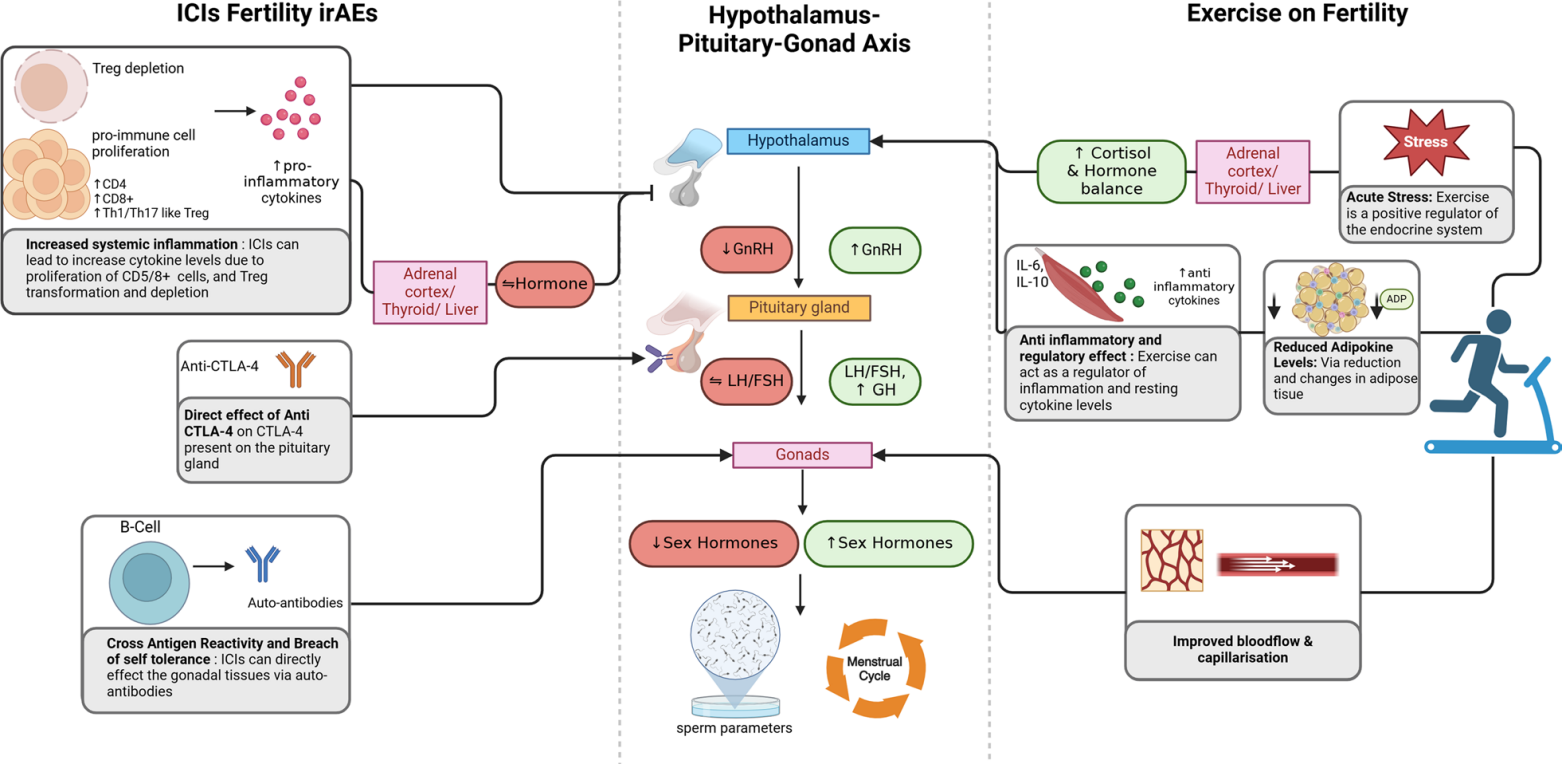
Potential mechanisms of immune checkpoint inhibitors (ICIs) and exercise on reproductive health. This diagram outlines the potential mechanisms through which ICIs and exercise influence the hypothalamus–pituitary–gonad (HPG) axis and fertility. ICIs may contribute to fertility-related immune-related adverse effects (irAEs) by causing Treg depletion, increasing systemic inflammation, and directly affecting pituitary and gonadal tissues, leading to hormone imbalances and potential gonadal dysfunction. Additionally, ICIs might trigger auto-antibodies that further impact gonadal tissues. In contrast, exercise may positively influence fertility by reducing stress, balancing cortisol and other hormone levels, exerting anti-inflammatory effects, and improving blood flow and capillarization, all of which support hormonal regulation and reproductive health. Created with https://www.BioRender.com

Furthermore, with the advancements in cancer treatments and medical technologies, the survival rates of cancer patients are increasing [86]. However, the long-term effects of the treatments can lead to infertility and temporary or permanent decrease in gonadal function [86]. Although earlier research has shown that exercise is helpful for both male and female infertility, there are currently insufficient clinical trials to support the idea that exercise can help reduce gonadotoxicity from cancer treatments. In the following section, an overview of the potential mechanisms through which exercise influences both endocrine-related fertility and gonadal function is provided. These mechanisms are comprehensively summarized in Fig. 2.

### Effects of exercise on endocrine-related fertility impairments

Different types of exercise have different impacts on endocrine function and immunological markers. A previous review article has summarized that a single bout of endurance exercise enhances cortisol levels, but ongoing endurance exercise increases basal cortisol due to HPA axis adaptation, with equivalent peaks in growth hormone reported [16]. On the other hand, resistance exercise causes minor HPA axis activation after a single session, but frequent resistance exercise reduces inflammation and lowers resting cytokine levels [16]. Hypophysis has been mentioned to disrupt the pituitary gland leading to structural changes in HPG axis [18] and exercise could influence the role of this axis in terms of sexual and reproduction health based on type, intensity, and the duration of the exercises. However, excessive exercise can have a deleterious effect on the HPG axis in both male and female [87]. Previous studies reported that testosterone peaks with maximal load exercises [88, 89] and can boost the estrogen in young women [90], whereas prolonged, intense endurance exercise depletes these hormones and disrupts hormonal balance; in contrast, moderate duration exercise efficiently raises endocrine hormone.

A single bout of endurance exercise above 60% VO2max stimulates the release of IL-6, which strongly activates the HPA axis and increases cortisol, GH, and prolactin levels as part of the stress response [91]. Over time, with regular endurance exercise, the body adapts to repeated stress, showing a moderate HPA response and higher baseline IL-6 and cortisol, indicating an adjustment to repeated stress [92]. Both single and regular sessions of exercise increase GH similarly. These adaptations illustrate how the endocrine system adjusts to sustained exercise stress [16]. Conversely, a single bout of resistance exercise triggers mild activation of the HPA axis, with the degree of activation influenced by exercise intensity and volume. It also raises catecholamine responses and TNF, a pro-inflammatory marker. Intense eccentric (muscle-lengthening) exercises appear to elevate IL-6, suggesting a potential link between muscle damage and IL-6 release, though the broader effect of resistance exercise on IL-6 are still under investigation [93]. Additionally, resistance exercise further enhances GH levels proportionally to exercise intensity, although regular training does not seem to affect baseline GH levels. Similarly, repeated resistance exercises do not significantly elevate baseline cortisol levels [94]. In terms of hormonal changes, a previous study reported that moderate-intensity exercise improves ovulatory function in young females by enhancing follicular sensitivity to FSH and LH, as indicated by a significant reduction in day 3 for estrogen, LH, and FSH, while increasing in day 21 [95]. In contrast, moderate exercise can enhance fertility issues in obese males by raising FSH to support sperm production and testosterone levels, stimulating LH to aid testosterone production, and reducing estradiol to balance hormone levels [96].

T1DM has been reported to be one of the adverse events from irAE that can disrupt the fertility. Previous meta-analysis has mentioned that exercise has been proven to be a non-pharmacological strategy for managing T1DM by promoting chronic glycemic control, enhancing insulin sensitivity and stimulating muscle glucose uptake [97]. The American College of Sports Medicine (ACSM) has released guidelines for prescribing the exercise in T1DM [**?**]. This guideline recommends individuals with T1DM exercise for 20–45 min at an intensity of 40–60% of their maximal oxygen consumption for 5–7 days per week, or daily at low to moderate intensity. However, a few precautions need to be taken for this population because high insulin levels during and after exercise can cause hypoglycemia, and low insulin levels may result in hyperglycemia and ketosis [97**?**].

### Effects of exercise on gonadal function and reproductive health

The impact of various exercise regimens on hypogonadism vary. In males, endurance exercise is proven to boosts the expression and activity of endothelial nitric oxide synthase (eNOS), leading to sustained increases in nitric oxide (NO) [98]. NO plays a key role in signaling, including relaxing smooth muscles and aiding vasodilation, which is crucial for penile erection (10). In females, particularly with PCOS, moderate endurance exercise significantly improves female reproductive health, increasing ovulation rates by 43.3% and restoring menstrual cycles by 56.7% [99]. The modulation of HPG axis and regulation of HPA axis might help improving these issues [17]. Furthermore, irAE treatment might induce testicular damage, affecting spermatogenesis. Existing studies have found that moderate-intensity endurance exercises such as cycling, walking, hiking can increase the sperm concentration by 25% [100], whereas resistance exercise was reported to improve sperm mortality, count, and morphology in male [101]. A preclinical study also found that endurance exercise can lead to testicular health by improving the testis morphology, reducing germ cell apoptosis, and alleviate the rise of inflammatory cytokine IL-6 after 10 weeks of swimming exercises [102]. Exercise has numerous benefits for gonad health, including improved testicular health and ovulation. Continuous moderate-intensity exercise reduces inflammation by balancing pro-inflammatory and anti-inflammatory reactions, which promotes Leydig cell activity, testosterone production, and overall testicular health. In women with PCOS, exercise helps generating a normo-androgenic environment by lowering inflammation, which can promote hyperandrogenism and interrupt menstrual and ovulation cycle. Both exercises have proven to reduce inflammatory markers IL-6, TNF-a, and CRP.

## Current approaches to immunotherapy-induced toxicity: challenges and future directions

Fertility preservation is crucial for patients undergoing immunotherapy, but limited knowledge of immune-related gonadotoxicity complicates guidance which makes pre-treatment fertility counseling and post-treatment family planning guidance challenging [103, 104]. The American Society of Clinical Oncology recommends embryo or oocyte cryopreservation for females and sperm cryopreservation for males as the gold standard, though these require hormonal stimulation, which may impact cancer patients [105]. Embryo cryopreservation is highly effective but has ethical challenges [103], while oocyte cryopreservation, with 30–45% efficiency, requires 2–3 weeks of preparation, making it unsuitable for urgent cases [106]. Alternatives include ovarian tissue cryopreservation, which offers over 90% ovarian function recovery but risks cancer cell reimplantation, and GnRH agonists (GnRHa), which provide endocrine protection but are not yet standard due to potential complications [106]. The National Comprehensive Cancer Network (NCCN) advises birth control during treatment and for 5 months after, with women avoiding conception for 6 months and men for 2 months posttreatment [104]. Lastly, ovarian stem cell differentiation into oocytes in vitro shows promise but remains experimental and technically demanding [106].

Recent studies on ICIs have reported various adverse effects on the immune system, hormones, and glands, yet none have directly addressed reproductive health. This is particularly concerning as many patients undergoing immunotherapy are young and of childbearing age, making fertility and pregnancy safety critical considerations. Despite the known risks of autoimmune side effects on endocrine functions, data on the impact of ICIs and gonadotoxic therapies on fertility are lacking, and the health-related quality of life in these patients remains insufficiently explored. So far most studies focus on older adults who are beyond the active age of fertility [65, 107]. Additionally, some studies combined outcomes from cancer and non-cancer patients, which can be problematic since cancer patients undergo more complicated situations due to the differing mechanisms of cancer treatment. Notably, ongoing studies registered on ClinicalTrials.gov include two studies specifically focused on fertility and pregnancy outcomes in cancer patients of childbearing age (NCT06242522 and NCT05429138). These studies aim to evaluate the safety of immunotherapy by assessing anti-Müllerian hormone levels, antral follicle count, semen quality, and the time to pregnancy after discontinuing contraception [108, 109]. Additionally, an observational cross-sectional study (NCT03946007) is investigating endocrine function in patients aged 18 years and older with melanoma, measuring gonadal and pituitary function in the blood 3 years after receiving ICIs, although results are not yet published [**?**]. Despite the high potential of exercise in mitigating infertility, no clinical study has examined the effects of exercise intervention or physical activity on reproductive health in cancer patients treated with ICIs. There are a few clinical trials registered involving exercise interventions during ICI treatments (NCT03171064, NCT06152926, NCT05358938) [110–112]. Although these studies do not focus on fertility outcomes, they aim to assess the safety, feasibility, and effectiveness of physical activity for cancer patients undergoing ICIs treatment. The insights gained from these trials could lead to the development or modification of practices that encourage physical activity and exercise for those receiving cancer treatment.

Future studies should investigate the mechanisms by which ICIs induce autoimmune reactions that impair ovarian and testicular function, as well as the potential impact of exercise on ovarian insufficiency and sperm production in cancer patients. Prioritizing the vigilant monitoring of endocrine function in these patients is essential to understand the long-term implications on reproductive health. Additionally, research should focus on the optimal timing of exercise interventions, both before and during ICIs treatment, to mitigate gonadotoxicity and prevent oncofertility issues. Comprehensive studies are necessary to explore the extent of reproductive risks and to develop strategies for fertility preservation. These studies will be critical for improving patient counseling as the use of ICIs continues to expand.

## Conclusion

Evidence suggests that ICIs treatment in cancer patients may result in gonadotoxicity, with a notable gap in data concerning female patients. Exercise, as a non-invasive intervention, has demonstrated protective effects against fertility-related toxicities. The efficacy of various exercise modalities, with differing volumes and intensities, in enhancing reproductive health is well established in both male and female populations. Therefore, future research should prioritize investigating the specific impact of exercise interventions on reproductive health in this demographic and elucidate the underlying mechanisms through which exercise confers protection against fertility impairment.

## Data Availability

Not applicable.

## Abbreviations

ACSM: American College of Sports Medicine
AGEs: Advanced glycation end-products
AMH: Anti-Mullerian hormone
APCs: Antigen-presenting cells
BATF: Basic leucine zipper ATF-like transcription factor
BBB: Blood–brain barrier
B cells: Pancreatic beta cells (responsible for insulin production)
BTB: Blood–testis barrier
CD4 +: Cluster of differentiation 4-positive T cells
CD8 +: Cluster of differentiation 8-positive T cells
CD80: Cluster of differentiation 80
CD86: Cluster of differentiation 86
CTLA-4: Cytotoxic T-lymphocyte associated protein 4
eNOS: Endothelial nitric oxide synthase
Fc*γ*Rs: Fc gamma receptors
FDA: Food and Drug Administration
FSH: Follicle-stimulating hormone
GnRH: Gonadotropin-releasing hormone
GnRHa: Gonadotropin-releasing hormone agonists
HP(X) axis: Hypothalamus–pituitary–organ axis
HPG axis: Hypothalamus–pituitary–gonadal axis
HPO axis: Hypothalamus–pituitary–ovarian axis
HPT axis: Hypothalamus–pituitary–testicular axis
ICIs: Immune checkpoint inhibitors
IFN-*γ*: Interferon-gamma
IL-1, IL-2, IL-6, IL-8: Interleukins 1, 2, 6, and 8
irAEs: Immune-related adverse effects
LH: Luteinizing hormone
MHC: Major histocompatibility complex
NCCN: National Comprehensive Cancer Network
NO: Nitric oxide
OSM: Oncostatin M
SPARC: Secreted protein acidic and rich in cysteine
PCOS: Polycystic ovary syndrome
PD-1: Programmed cell death protein 1
PD-L1: Programmed death ligand 1
PD-L2: Programmed death ligand 2
T1DM: Type 1 diabetes mellitus
TCR: T cell receptor
TFR: Follicular regulatory T cells
TFH: T Follicular helper cells
TME: Tumor microenvironment
TNF-*α*: Tumor necrosis factor-alpha
Tregs: Regulatory T cells
Zap70: Zeta-chain-associated protein kinase 70
